# Assessment of digital risks in child and adolescent mental health services: A mixed-method, theory-driven study of clinicians’ experiences and perspectives

**DOI:** 10.1177/13591045221098896

**Published:** 2022-05-06

**Authors:** Alex Lau-Zhu, Ciorsdan Anderson, Matthew Lister

**Affiliations:** 1 Medical Sciences Division, Oxford Institute for Clinical Psychology Training and Research, 6396 University of Oxford, UK; 2 Division of Psychiatry, Department of Brain Sciences, Imperial College London, UK; 3 Child and Adolescent Mental Health Services, 8955 Oxford Health NHS Foundation Trust, UK

**Keywords:** Digital risk, online safety, social media, child and adolescent mental health services, COM-B, youth mental health

## Abstract

Children and adolescents in the UK spend increasingly more time in the digital world, raising societal fears about digital risks in this age group. Professionals are not always aware of the ever-developing research or guidance available around digital safety. This gap underscores the need to understand current experiences and determinants of digital risk assessment, including clinicians’ views on barriers and facilitators. A mixed-method design was used. Fifty-three clinicians working in child and adolescent mental health services (CAMHS) in South England took part in a survey; of these 12 took part in semi-structured interviews. A psychological model of behavioural change (COM-B: capabilities, opportunities, motivation and behaviour) guided the analyses. Survey data revealed that clinicians showed awareness and concerns for several digital risk issues but there appeared to be gaps in their knowledge and practice. Interview data revealed different factors influencing staff enquiry about digital risks in CAMHS. These included aspects of capabilities (knowledge and skills), opportunities (resources, organisational context and empowerment of youth), and motivations (habit change, emotional experiences, and professional identity/role). Targeting both staff-level and organisation-level barriers to digital risk assessments in CAMHS is crucial. This study informs service improvement to ensure that children and young people safely navigate the digital world.

The Internet plays an important role in young people’s lives. The majority of children and adolescents in the UK have their own tablets or smartphones and they also spend increasingly more time online (Ofcom, 2020), raising societal fears about digital risks in this age group (Odgers & Jensen, 2020; UNICEF, 2017). Currently, firm conclusions about the negative effects of screen-based activities on young people’s socio-emotional developmental and mental health are hampered by methodological issues within research (Dickson et al., 2018), including for controversial areas such as screen time (Orben, 2020). Nevertheless, practitioners understandably remain concerned by the emerging evidence (Dickson et al., 2018), particularly in areas associated with high levels of clinical risks, including self-harm (Biernesser et al., 2020), cyberbullying (Bottino, Bottino, Regina, Correia, & Ribeiro, 2015), and problematic sexual attitudes and behaviours (Horvath et al., 2013).

Several UK-based authoritative bodies have recently issued guidance on digital risks in youth (Livingstone et al., 2017; NSPCC, 2019; Viner, Davie, & Firth, 2019), all of which point to a critical need for professionals to understand the emerging evidence base within their practice. Most research on digital risks is on the impact on youth. Research on clinicians thus far has been largely restricted to their views on digital interventions (Cliffe, Croker, Denne, & Stallard, 2020), digital consultations (Bhardwaj et al., 2021), and internet safety education (Moreno, Egan, Bare, Young, & Cox, 2013) but not on digital risk assessments, despite their active role in prevention, intervention and safeguarding.

In this rapidly developing field, we note that several terms are used interchangeably, including – but not limited to – digital risk (OECD, 2021), digital dangers (Palmer, 2015), online risk (Livingstone, Lievens, & Carr, 2020), online safety (NSPCC, 2019), online harm (Woodhouse, 2021), Internet safety (Livingstone et al., 2017), cybersecurity (Quayyum, Cruzes, & Jaccheri, 2021), and so forth. Our rationale for choosing “digital risk” was pragmatic: “risk” is the language used by clinicians to denote common forms of harm to patients (e.g., risk of suicide or neglect) and “digital” is a more encompassing term that covers both online and offline activities (i.e., using digital devices). Terminology and definitions are likely to consolidate with further intersectoral collaboration spanning science, law, health, education, and technology.

## Local context

Oxford Health is an NHS Foundation Trust in South England looking to develop guidance on digital risks for working with young people. Child and adolescent mental health services (CAMHS) in the UK are expected to offer advice and consultation around appropriate support when online behaviours can be detrimental to a young person’s development.

Professionals, parents and young people, however, are not always aware of the ever-developing research or guidance available around digital safety. Anecdotal accounts suggest a lack of confidence given the generational gap between clinicians and young people. It is also unclear whether online activity is appropriately covered in routine risk assessments. This gap underscores the need to identify current experiences and determinants of digital risk assessment, including clinicians’ views on barriers and facilitators.

While our focus is on one organisation, clinical experience and discussions with colleagues indicate that this is a topical area that holds relevance nationally in the UK (and internationally) and across statutory and tertiary sectors.

## A behaviour change approach

Psychological theories play an important role in understanding and informing behavioural changes to guide the development of high-quality interventions. An elegant pan-theoretical model (Michie, van Stralen, & West, 2011) posits that components associated with capabilities (C), opportunities (O) and motivations (M) are necessary for behaviour (B) to occur (Supplementary Material 1). A “diagnosis” of the determinants of the behaviour following this COM-B model can be made to identify intervention targets. Capabilities include internal factors such as knowledge and skills; opportunities include external factors such as physical (e.g., infrastructures) and social possibilities (e.g., organisational culture and implementation climate); and motivations include automatic (e.g., habit and emotions) as well as deliberate processes (e.g., beliefs and intentions) that would energise behaviour. Such components are informed by psychological constructs synthesized across 19 major behaviour-change theories, an approach coined as the Theoretical Domain Framework (TDF) (Atkins et al., 2017) (Supplementary Material 2).

The COM-B analysis can also help identify corresponding intervention and policy approaches to support the desired behavioural changes (Supplementary Material 1). For example, a lack of skills identified could be targeted via training, with regulation flagging this training up as compulsory; habit formation could be targeted with environmental restructuring to add reminder cues, which can be further enhanced with regular communication promoting this desired habit; the lack of social influences could be targeted by making role models more available, further supported by a widely disseminated professional guidance.

The COM-B model is increasingly used in healthcare research. This model has informed clinical practice to change clinicians’ behaviours in areas such as risk communication (Bonner et al., 2014), health promotion (McDonagh et al., 2018), health assessments (Alexander, Brijnath, & Mazza, 2014), use of evidence base (De Leo, Bayes, Bloxsome, & Butt, 2021), and improved use of clinical resources (Jenkins et al., 2018). We are, however, unaware of research applying COM-B to understand what is needed for clinicians to perform risk assessments more effectively.

## Aim and questions

Our overarching aim is to understand clinicians’ experiences and perspectives in relation to digital risk assessment in CAMHS in Oxford Health and to inform service development in this area. This research is guided by the COM-B (and TDF) approach with emphasis on mapping determinants of behaviour.

Our specific questions were as follows:

1. What areas of digital risks in children and young people are clinicians concerned about and what areas are being assessed by clinicians?
2. What clinicians’ characteristics (demographic and professional factors) are associated with their concerns, assessments and knowledge of digital risks?
3. What are the barriers and facilitators for clinicians in assessing digital risks?
4. What recommendations (e.g., intervention and policy approaches) can be drawn for service developments?

## Methods

### General design

A mixed-method design was used. Two studies were conducted. In Study 1, an online survey was devised to map out clinicians’ views and experiences on digital risks and to explore the associations with participants’ characteristics. In Study 2, a subsample of clinicians was followed-up with interviews to explore their views on barriers and facilitators for assessing digital risks in CAMHS. Participation was voluntary and all participants provided informed consent. The project was approved and registered by Oxford Health NHS Foundation Trust Quality and Audit Team as service evaluation, and thus no ethical approval was needed.

Additional method details can also be found on Supplementary Material 3.

### Study 1

#### Participants

All team managers in CAMHS within Oxford Health were contacted to invite clinicians within their teams to take part in a short online survey. Survey data were collected between September and December 2020.

#### Design and procedure

An online survey (Supplementary Material 4) was designed using the Joint Information Systems Committee (JISC) Online Survey tool. Participants were told that their individual responses would remain confidential and that the survey would take approximately 15 min to complete. The survey questions were devised by the authors in consultation with clinicians and researcher working in youth mental health.

Survey questions were divided into three main sets. The first set asked about demographics and professional characteristics. The second set asked about concerns and assessment on key areas of digital risk with four response options (never, seldom, sometimes and often). For all these areas, clinicians were asked about their own frequency of concerns as well as their perception of both young people’s and parents’/caregivers’ frequencies of concerns. The third set was intended as a brief test of knowledge on the issue of digital risks. Clinicians were asked whether they were aware of any guidelines on the topic and whether they knew of eight apps (used by young people) which pose some level of risks according to NSPCC (National Society for the Prevention of Cruelty to Children). Finally, a link to resources on online safety by NSPCC was included.

#### Analysis

Descriptive statistics were used to summarise demographics and professional characteristics. Frequencies of concern and frequency of assessments per area of digital risks were totalled across clinicians. Knowledge was assessed as whether clinicians were aware of guidelines on digital risks; the number of apps that clinicians were able to correctly identify; and the number of apps that clinicians were able to recognise as somewhat risky for young people. Frequency responses indicating “often” and “sometimes” were grouped into “high” frequency and “seldom” and “never” into “low” frequency.

Fisher’s exact tests were used to explore associations between digital risk concern/assessment/knowledge and participant characteristics. The Benjamini-Hochberg procedure to control for false rate discovery (*q* < .05) was applied given the large number of tests. All statistical analyses were performed using SPSS version 25 (IBM, 2017).

### Study 2

#### Participants

Clinicians who completed Study 1 were invited to leave their contact details if they wished to be interviewed. Participants were told that the findings will help develop strategies to better identify and manage digital risks in CAMHS. Interviews took place between November 2020 and January 2021.

#### Design and procedure

A topic guide (Supplementary Material 5) was jointly developed and piloted by the study authors and subsequently reviewed based on feedback from clinicians working in CAMHS. We began with a broad definition of “digital risks” as “*behaviours involving the use of digital devices, the Internet and/or social media that pose potential harm to self, to others, or from others, in young people that you work with*”. Interview questions explored clinicians’ experiences of assessing digital risks with young people; factors (personal and professional) perceived as influencing their experiences/views of assessing digital risks; and recommendations for service improvement. All interviews took place individually by videoconferencing. Each interview was between 30–60 minutes.

#### Analysis

Interviews were audiotaped and transcribed. Transcripts were subsequently analysed with thematic analysis (Braun & Clarke, 2006) using NVivo 12 (International QSR, 1999). The lead author (ALZ) read each transcript several times to ensure familiarization and then conducted the initial coding and provisional (sub)theme generation. All authors reviewed the final (sub)themes and interpretation of the data to ensure credibility and coherence.

## Results

### Study 1

#### Participant characteristics

Fifty-three clinicians responded to the online survey (see Supplementary Material 6 for full details on demographics and professional characteristics). Respondents were primarily female, White and working full-time. There was an even split between parents and non-parents.

A wide range of work experiences were reported as indicated by the number of years since core qualification and number of years working for Oxford Health (ranging from less than a year to over 30 years). All core professions within CAMHS in the UK were represented (with the majority identifying as psychologists or nurses). All CAMHS services within Oxford Health in Oxfordshire were also represented. The majority of clinicians worked within local, generalist CAMHS and a county-wide specialist neurodevelopmental service. Most clinicians reported having a weekly caseload of 4–9 direct or indirect cases (i.e., working through families, schools or other professionals).

#### Digital risk concerns and assessment

**Figure 1.**
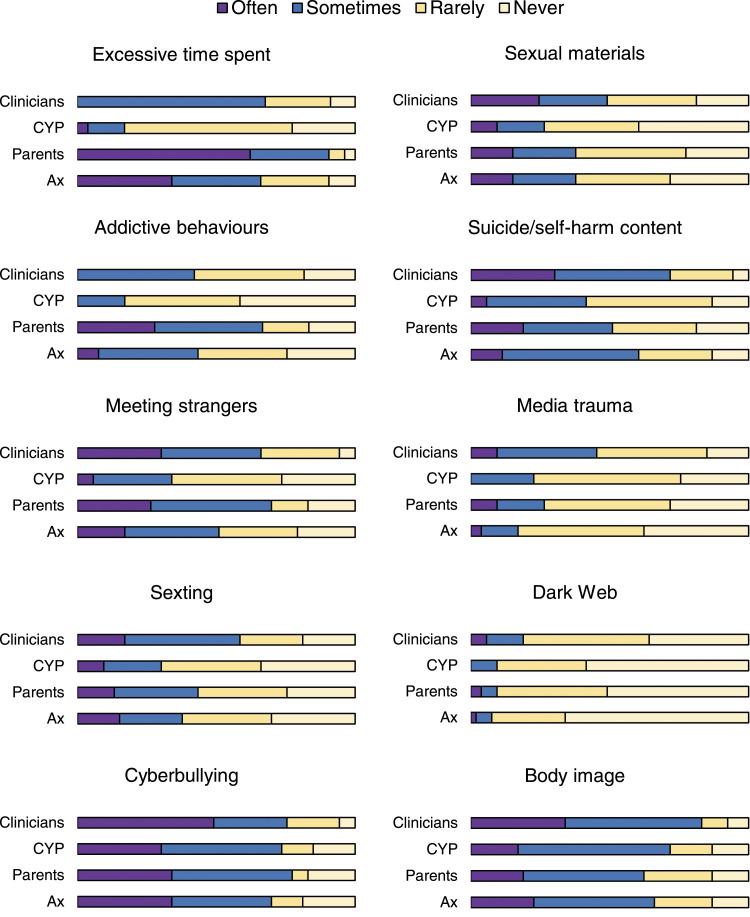
Frequency of concerns on key areas of digital risks as reported by clinicians. Note. Clinicians = how often *clinicians* are concerned about this area of digital risk; CYP = how often clinicians perceive that *children and young people* are concerned about this area of digital risk; parents = how often clinicians perceive that *parents/caregivers* are concerned about this area of digital risk; ax = how often do clinicians *assess* this area of digital risk.

Six areas of digital risks were rated by clinicians as of “high” concern (i.e., with at least 50% of clinicians endorsing “often” or “sometimes”). These were time spent; meeting strangers; sexting; cyberbullying; suicide/self-harm; and body image (Figure 1). The remaining areas not rated as such were addictions, sexual materials (e.g., pornography), media trauma, and the dark web. Clinicians’ frequency of concerns was significantly associated with their frequency of assessments of all digital risk areas, except for media trauma (see Supplementary Material 7 for detailed results).

Clinicians’ frequency of digital risk concerns was significantly associated with their perception of young people’s concerns in all areas except for three (time spent, meeting strangers and addiction). Clinicians’ frequency of concerns were significantly associated (accorded) with their perception of parents/caregivers’ concerns in all areas.

#### Brief test of clinicians’ knowledge

Only one third of clinicians (*n* = 19; 35.8%) reported being aware of any existing guidelines. While clinicians on average identified most of the eight apps correctly (*M* = 6.17; *SD* = 1.82), they recognised only approximately half of these as somewhat risky (*M* = 3.74; *SD* = 2.03).

#### Associations between clinicians’ characteristics and clinicians’ digital risk concerns, assessments and knowledge

Digital risk *concerns* showed mostly no significant associations with any clinicians’ characteristics, with two exceptions. More *years qualified* was significantly associated with more clinicians’ concerns about sexting and sexual materials. Working in services with *Tier 4* provision was also significantly associated also with more concerns also about sexting (Tier 4 services in the UK CAMHS system typically funded to offer more specialist, county-wide support and have closer links to youth justice and exploitation services). No significant associations were found with any other areas of digital risks. No significant associations were found with age or parenthood.

Digital risk *assessments* showed a significant association with only one clinicians’ characteristic. Specifically, more *years qualified* was significantly associated with more assessments of time spent, sexting; exposure to sexual materials; body image; suicide/self-harm; and meeting strangers. No significant associations were found with any other area of digital risks. No significant associations were found with age, parenthood, or CAMHS tiers.

Finally, awareness of guidelines was significantly associated with more *years qualified* and with *being a parent*. No significant associations were found with age or CAMHS tiers or with knowledge about risky apps.

See Supplementary Material 7 for detailed results.

### Study 2

#### Participant characteristics

Twelve clinicians took part in the interviews. Nine identified as female and the rest as male. A range of professions were represented: five clinical psychologists, a forensic psychologist, a forensic psychiatrist, a child and adolescent psychiatrist, a nurse, a social worker, an educational wellbeing practitioner, and an assistant psychologist. There was an equal split in those working in CAMHS services with and without Tier 4 provision.

#### Themes and subthemes

**Figure 2.**
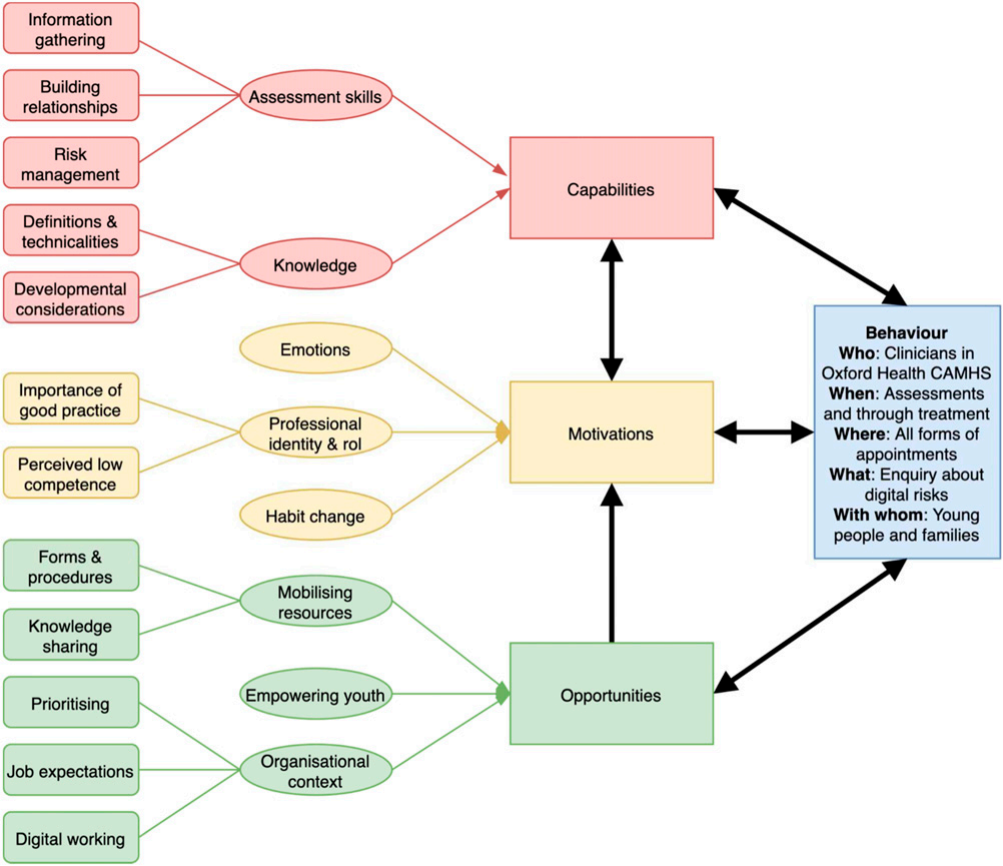
Schematic of Themes and Subthemes following the COM-B Model. Note*.* A generic COM-B model (Michie et al., 2011) was expanded with current themes and subthemes.

Eight themes and 13 subthemes were identified, which corresponded to two capability components, three opportunity components and three motivational components within the COM-B model (Figure 2). A more detailed summary of the relationships between COM-B components (and corresponding TDF domains linked to specific theory-driven psychological constructs) can be found in Supplementary Material 8. Additional illustrative quotes can be found in Supplementary Material 9.

##### Assessment skills (capability)

Clinicians described an approach to *information gathering* that needed to be both narrow and broad. While some clinicians waited for a concern before exploring further, the need for direct question was also raised: “you just can’t pick it up … asking the question 150 times until you get one ‘yes’ … that’s worth asking the question” (participant 10). The use of formulation was raised as a guide to assessment, placing emphasis on the broader context, including school and family factors and areas of strengths. Thus, sensitively involving parents was highlighted as a key issue. Overall, a semi-structure approach was favoured that included general enquiries followed by prompts. Given the potential sensitive nature of this topic, an emphasis was placed on *building relationships* with the young person, and getting to know the young person (participant 3). Finally, clinicians wanted to be skilled in *risk management* beyond assessments, as the former is perceived as harder (participant 8). Clinicians often expressed feeling unable to provide adequate advice for young people in making changes and for parents in supporting these changes.

##### Knowledge (capability)

Clinicians described gaps in their knowledge of *definitions and technicalities*. Many expressed not knowing the whole “spectrum of risks*”*, including areas they never thought they would ask about (participant 2). Lack of knowledge was described about the specifics of how an app works – “bamboozled by the technical side of it” (participant 6). Potential ambiguities were also highlighted regarding the umbrella term “digital risks”. Clinicians expressed the need to be thoughtful of *developmental considerations*. Clinicians would like to know more about the general prevalence of digital risks (participant 12) and what falls within normative risk-taking (participant 2). There is a desire for an overarching framework including typology/principles rather than just learning technicalities, as these can change rapidly (participant 6), as well as better understanding vulnerability and maintenance factors (participant 11).

##### Professional identity and role (motivation)

Clinicians described the *importance of good practice*. Considering digital risks was seen as “due diligence” (participant 3) – “it won’t take long ‘cause it’s not relevant or it will take long and it will be a bloody good job that we’ve done it” (participant 1). Unanimously, the need for education, training and regular updates was stressed, as it is about “practice catching up with the world outside” (participant 6). Many also raised a sense of *perceived low competency* and generally “not feeling equipped” (participant 12). There is also a perceived “generational gap” – “I’m at an age now where I think ‘oh, God, do I need to know anymore?’ … their knowledge … it’s more embedded … whereas mine on the outskirts?” (participant 8).

##### Habit change (motivation)

Clinicians explained challenges associated with “moving from ‘oh this is a good idea’ to … actually put something in place” (participant 1). Clinicians explained that it can be easy to forget because “there’s so many things to remember that it can be really hard to remember all of them” (participant 2). Suggestions proposed included the role of practice (participant 2) and having reminders (participant 5).

##### Emotional experience (motivation)

The issue of digital risks brought up a range of emotions – the “speed of things ….I find so daunting” (participant 8). “Taboo” subjects, especially those involving sex, seem to bring up anxieties (participant 12). Feelings of helplessness and frustration were described when not knowing how to manage risks (participant 9). Several clinicians wondered if a “big incident” is needed to prompt further awareness of digital risks as “motivation is emotional” (participant 1).

##### Mobilising resources (opportunity)

A range of suggested improvements for *forms and procedures* were made. A lack of standardised “formal template” where digital risks are included was mentioned (participant 11), either in paperwork or within CareNotes (an electronic recording system). A clinician commented that the electronic system presents “an adult risk assessment that didn’t really make sense for young people”, for example, with issues such as risk of falls (participant 5). A “simple checklist” (participant 6) or a “short and compact screening” form (participant 4) with “little prompts” (participant 1) was suggested to aid digital risk assessments. Suggestions were also made for improving *knowledge sharing.* They wanted more “joint-up thinking about what’s currently going on” (participant 3). These included using of existing structures such as team meetings to share information, with some clinicians preferring “regular” and “practical” training (participant 8) while others just “brief” updates (participant 2). Many wanted clarity around “where to go to” for help (participant 6), including safeguarding teams, nominated digital risk champions and/or clinical supervisors.

##### Organisational context (opportunity)

Issues around *prioritising* were discussed, with senior managers “being on board” seen as critical (participant 9). Clinicians pointed out some gaps in the organisation as a whole on this topic, for example, the lack of digital risks in induction/current training or broader conversations on this topic beyond CAMHS. However, some caveats were raised, for instance, that “it can be a more of a corporate thing … kind of top down or like someone’s hobbyhorse” (participant 1), alongside competing demands – “there were so many things we could add in … it’s hard” (participant 5). There was also a desire for clarity on *professional expectations*. Clinicians commented on “a lack of “national/cross-agency agenda” and “professional practice guidance” (participant 6), leading to variability in practice. Recent Organisation-level uptake in *digital working,* due to the safety restrictions imposed by the COVID-19 pandemic, has meant that there is now more “awareness” of digital risks (participant 8) and opportunities to use young people’s “language” (participant 7).

##### Empowering youth (opportunity)

Clinicians expressed the need to put young people’s views and experiences at the centre of this topic. Understanding young people’s views on digital risk assessments was seen as critical, for instance asking “if you were coming to CAMHS, how would it be if someone asked you about your social media usage?” (participant 3). Challenges were discussed around how to best enable young people to share, as they often “don’t feel being understood by parents” (participant 12) and needed to be “brave” to share (participant 2). Some suggested for “a questionnaire that the young person fills out while they’re in the waiting room…just like other routine outcome measures” (participant 11). A more active role within a broader service-user engagement strategy was proposed, because it is a“…whole world that young people know a lot more about than older people, and its often older people planning and organising services and less involved in social media”. Suggestions were made for young people to be involved in training/educating clinicians to create a genuine sense of “constant conversation back and forth” that “could potentially have more impact” (participant 5).

## Discussion

### Key findings

Overall, survey data provided a quantitative snapshot of clinicians’ views and experiences on digital risks showing variability in practice, and interview data further revealed a theory-driven core set of behavioural determinants (by identifying themes) of current practices in digital risk assessments (as perceived by clinicians).

Here we summarise the key results as brief answers to each of our four research questions (see *Aim and Questions*) in turn:

1. Clinicians showed concerns and assessed for a range of digital risks areas (Figure 1) although with some gaps (i.e., gaming and Internet addiction, pornography, media trauma and the dark web); they also showed awareness of common apps used by young people but not of all possible risks associated or of existing guidelines; and their concerns were in line with parents’ concerns but not always with young people’s concerns;
2. Very few associations were found with clinicians’ characteristics, including age and parenthood, suggesting that these individual characteristics may play less of a role in clinicians’ practices on digital risks than it is assumed by the perceived “generational gap”;
3. Factors in all domains of capabilities, opportunities, and motivations (Figure 2) were found as relevant for clinicians, most of which represented barriers to routine enquiry of digital risks in CAMHS;
4. These factors represent potential targets for behavioural-change interventions and policy approaches (Michie et al., 2011), which we elaborate below (also see Supplementary Material 10).

### Implications for service improvement

#### Intervention functions

Interview data echoed survey results in the need for interventions targeting multiple factors and at multiple levels. Clinicians have suggested a wide range of potential behavioural-change techniques for intervention development (details which can be found in Supplementary Material 8). At the staff level, there is a role for *education* and *training* to increase capabilities (knowledge and skills) and motivations (e.g., confidence). This is likely to produce maximal impact when it is responsive to staff and service needs (i.e., considering each subtheme identified) while bearing in mind that a one-off “tick box exercise” is unlikely to cover the breadth and evolving nature of the issues. Interventions taking the form of *environmental restructuring* (e.g., new assessment resources and processes) and *modelling* (e.g., knowledge sharing with supervisors and colleagues) can play important roles in engaging more “automatic” or “unconscious” processes driven by external factors (Marteau, Hollands, & Fletcher, 2012), especially when “reflective” or “conscious” motivations are limited in fully driving behavioural change. For instance, clinicians showed conscious awareness of issues – it was perceived as important for their professional identity – but they still identified significant barriers and gaps in their practice of digital risk assessments.

Beyond staff-targeted interventions, clinicians suggested the need for broader systemic changes through services as well as the whole Trust. Implementation science theories stress the importance of concurrently targeting staff and organisational-level determinants (Williams & Beidas, 2019). The limited benefits of training-only approaches on *behavioural change* – by just targeting staff – can be illustrated by the popular yet ineffective use of unconscious bias training, which typically consists of one-off, stand-alone educational programs delivered to individuals to increase awareness of unconscious biases that impact minoritised groups based on ethnicity, disability, and so forth (Forscher et al., 2019). In addition to the approaches already described, a shift in organizational culture is needed for *enablement*, including leadership and managers that encourage attention to digital risks (e.g., adding to the business agenda and promoting meaningful service-user participation). While changes in healthcare systems are typically perceived as slow, we have also witnessed how NHS services in the UK have continued to swiftly adapt to a worldwide pandemic through concerted efforts throughout organisations.

#### Policy categories

While some clinicians were aware of guidelines on digital risks, there is a clear need for developing *guidance* as an organisation to ensure consistent and best evidence-based practice for individual staff, teams and services as well as for sustainable mutual learning. In the absence of a national, cross-sector guideline, recent guidelines developed by authoritative bodies (Livingstone et al., 2017; NSPCC, 2019; Viner et al., 2019) will be most informative. There is also a role of *regulation* to monitor and audit assessment practices and thus help evaluate the uptake of best practice and identify areas of challenge. *Environmental planning* would be crucial to ensure that forms, procedures and electronic systems facilitate timely information gathering and recording on digital risks. Regular *communication* to staff and teams should reflect priorities on digital risks while embedding them within a broader digital strategy. We note that Oxford Health has been recently named “Mental Health Global Digital Exemplar” (NHS England, 2021) based on its digital innovations. Associated funding and partnerships could be harnessed for further work in considering digital risks in clinical practice.

### Strengths and limitations

A key methodological strength of the study is the use of a psychological model to guide data analysis and interpretation, thus harnessing a theory-driven approach to behavioural change for maximal impact. The mixed methodology combining surveys with interviews helped develop a more comprehensive understanding of clinicians’ experiences and perspectives than either method only would have allowed. Professionals were closely consulted in the methodology to ensure the relevance of the questions. A wide range of key CAMHS professional backgrounds and service types were included.

Limitations must also be noted. The sample may have been biased by clinicians who are already interested in the topic so it is possible that a different pattern of results holds for those who did not participate, although making participation compulsory can raise ethical complexities. The sample size was modest for a survey study but standard for an interview study using thematic analysis for a rich dataset. Other potential areas of digital risks (e.g., radicalisation) were not included. Additional work is required to test whether the current findings generalise to other contexts, including CAMHS settings in other areas, services outside the UK and non-healthcare sectors. Crucially, not all stakeholders’ views were included, and in particular young people’s views of reporting on digital risks should be sought.

## Conclusion

**Figure 3.**
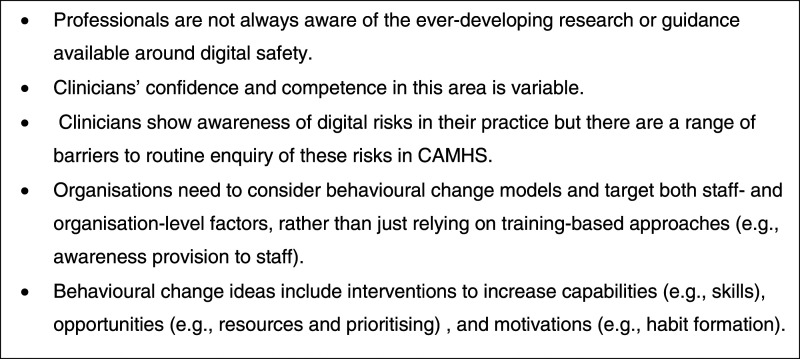
Key practitioner message.

What is needed for clinicians in CAMHS to embed routine enquiry of young people’s digital risks into their practice? We have summarised a key practitioner message (Figure 3). A behavioural change program that targets both staff-level and organisation-level barriers to digital risk assessments in CAMHS is crucial. Interventions and policies to support such programs should embrace psychological theories of behavioural change. This study informs service improvement and research – within healthcare systems and beyond – to ensure that children and young people safely navigate the ever-changing digital world.

## Supplemental Material

Supplemental Material - Assessment of digital risks in child and adolescent mental health services: A mixed-method, theory-driven study of clinicians’ experiences and perspectives Click here for additional data file. Supplementary Material for Assessment of digital risks in child and adolescent mental health services: A mixed-method, theory-driven study of clinicians’ experiences and perspectives by Alex Lau-Zhu, Ciorsdan Anderson, and Matthew Lister in Clinical Child Psychology and Psychiatry.
