# Supplementary material for: Assessment of digital risks in child and adolescent mental health services: A mixed-method, theory-driven study of clinicians’ experiences and perspectives
Source: Clin Child Psychol Psychiatry. 2022 May 6;28(1):255–69. doi: 10.1177/13591045221098896 (PMC9893305; doi:10.1177/13591045221098896)
Supplement: Supplemental Material - Assessment of digital risks in child and adolescent mental health services: A mixed-method, theory-driven study of clinicians’ experiences and perspectives [file sj-pdf-1-ccp-10.1177_13591045221098896.pdf]

Supplemental Materials 1

Figure S1

The Capability-Opportunity-Motivation-Behaviour (COM-B) Model at the Centre of the Behavioural Change Wheel

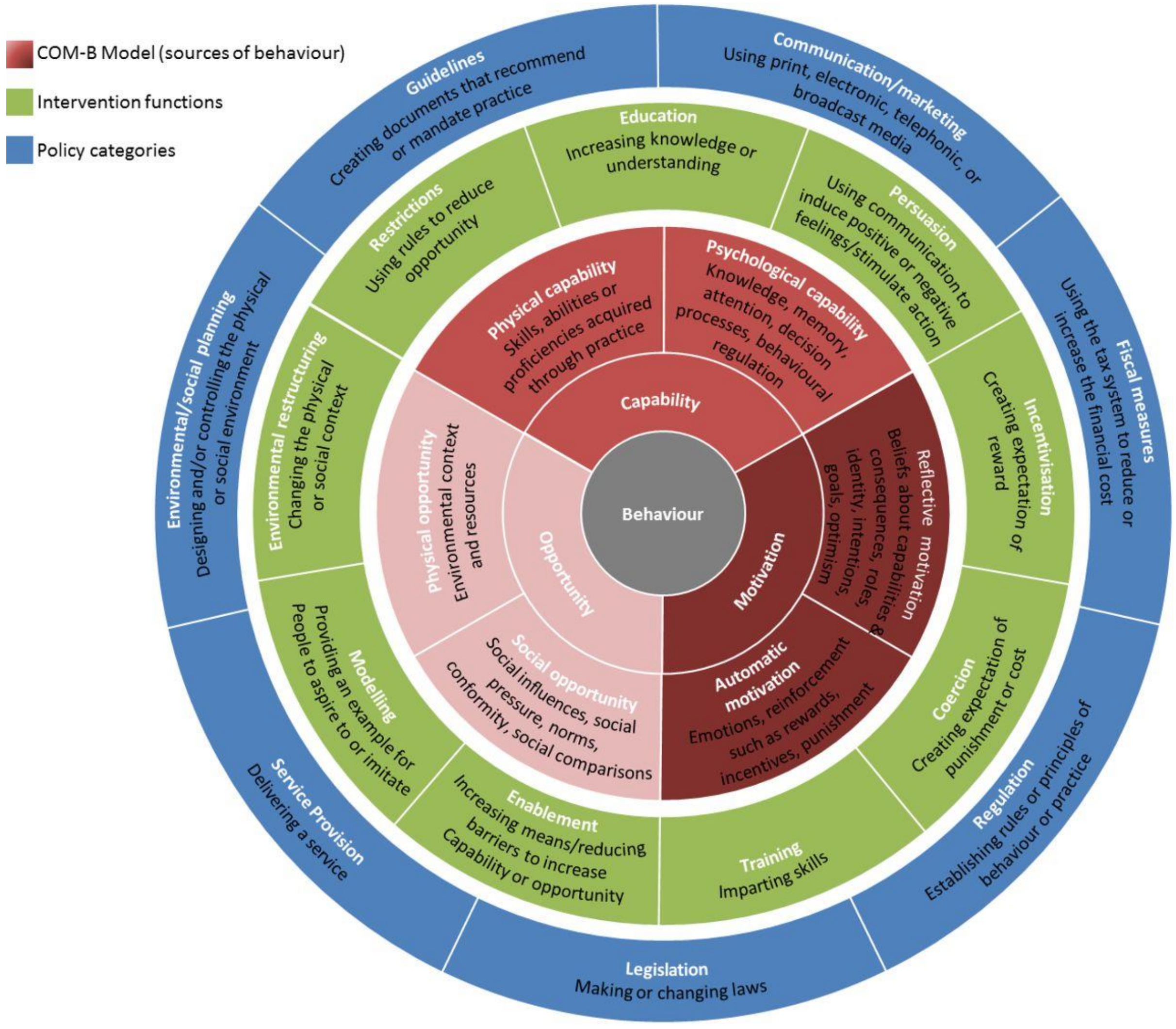

Note. *The COM-B model is depicted with associated interventions and policy categories, each with a brief description. Reproduced from McDonagh et al. (2020) which was adapted from Michie et al. (2010).*

Supplemental Materials 2

Table S1

Theoretical Domain Framework (TDF)

COM-B component	*TDF domain (definition)*	*Constructs*
Capability	*Knowledge** *(An awareness of the existence of something)*	Knowledge (including knowledge of condition /scientific rationale); procedural knowledge; knowledge of task environment
	*Skills** *(An ability or proficiency acquired through practice)*	Skills; skills development; competence; ability; interpersonal skills; practice; skill assessment
	Memory, attention and decision processes *(The ability to retain information, focus selectively on aspects of the environment and choose between two or more alternatives)*	Memory; attention; attention control; decision making; cognitive overload / tiredness
	Behavioural regulation *(Anything aimed at managing or changing objectively observed or measured actions)*	Self-monitoring; breaking habit; action planning
Opportunity	*Environmental context and resources** *(Any circumstance of a person's situation or environment that discourages or encourages the development of skills and abilities, independence, social competence, and adaptive behaviour)*	Environmental stressors; resources / material resources; organizational culture /climate; salient events / critical incidents; person x environment interaction; barriers and facilitators
	*Social influences** *(Those interpersonal processes that can cause individuals to change their thoughts, feelings, or behaviours)*	Social pressure; social norms; group conformity; social comparisons; group norms; social support; power; intergroup conflict; alienation; group identity; modelling
Motivation	*Social/professional role & identity** *(A coherent set of behaviours and displayed personal qualities of an individual in a social or work setting)*	Professional identity; professional role; social identity; identity; professional boundaries; professional confidence; group identity; leadership; organizational commitment
	*Beliefs about capabilities** *(Acceptance of the truth, reality, or validity about an ability, talent, or facility that a person can put to constructive use)*	Self-confidence; perceived competence; self-efficacy; perceived behavioral control; beliefs; self-esteem; empowerment; professional confidence
	Beliefs about consequences *(Acceptance of the truth, reality, or validity about outcomes of a behaviour in a given situation)*	Beliefs; outcome expectancies; characteristics of outcome expectancies; anticipated regret; consequents
	Optimism *(The confidence that things will happen for the best or that desired goals will be attained)*	Optimism; pessimism; unrealistic optimism; identity
	Intentions *(A conscious decision to perform a behaviour or a resolve to act in a certain way)*	Stability of intentions; stages of change model; transtheoretical model and stages of change
	Goals *(Mental representations of outcomes or end states that an individual wants to achieve)*	Goals (distal / proximal); goal priority; goal / target setting; goals (autonomous / controlled); action planning; implementation intention
	*Reinforcement** *(Increasing the probability of a response by arranging a dependent relationship, or contingency, between the response and a given stimulus)*	Rewards (proximal / distal, valued / not valued, probable / improbable); incentives; punishment; consequents; reinforcement; contingencies; sanctions
	*Emotion** *(A complex reaction pattern, involving experiential, behavioral, and physiological elements, by which the individual attempts to deal with a personally significant matter or event)*	Fear; anxiety; affect; stress; depression; positive / negative affect; burn-out

Note. The domains of the TDF are defined, mapped onto COM-B model, and specified in terms of associated psychological constructs. Adapted from Atkins et al. (2017). *Domains considered in the thematic analysis.

Supplemental Materials 3

Additional Methods

Study 1

Reminder emails to each team lead were sent twice. Psychological therapists were also emailed directly from a mailing list.

The survey questions were initially devised by ML and CA who have over 15 years of clinical experience in CAMHS in the UK, and by ALZ who has postdoctoral research experience in youth mental health. The survey was reviewed by clinicians and researchers working with youth and subsequently revised based on their feedback.

Ten key areas of digital risks were identified through the above consultation process: 1) excessive time spent online or on social media; 2) addictive behaviours (e.g., gaming, gambling); 3) meeting strangers online; 4) sexting; 5) abusive messages, cyberbullying or trolling; 6) exposure to sexual materials (e.g., pornography); 7) suicide or self-harm content (e.g., forums, images); 8) media traumas (e.g., viewing gruesome details of horrific events); 9) the dark web (e.g., buying illegal drugs); and 10) body image (e.g., exposure unrealistic standards and using forums/chats).

Some variables were recoded/dichotomised for this purpose. Age was recoded as "40 and below (millennials)" versus "above 40 (non-millennials)" based on the definition of millennials who experienced the "Internet explosion" (Pew Research Centre, 2019). Years qualified and number of correct apps identified were dichotomised based on median-splits.

Associations with gender and ethnicity were not explored, as the majority were identified as female and white. Missing data were not possible, as the survey used forced responses.

Study 2

Interviews were conducted individually by ALZ (a non-White, male clinical psychologist in training with over a decade of experience conducting research/clinical interviews).

The analytical approach can be described as deductive, semantic and essentialist/realist because coding was primarily informed by theoretical frameworks of behavioural change (COM-B and TDF); meaning extracted was at the explicit level; and our primary interest was in clinicians' perception of reality.

Supplemental Materials 4

Online Survey

INFORMATION SHEET AND CONSENT

Assessing digital risks in young people: Staff experiences and views

We invite you to take part in an audit exploring clinical staff experiences and views of assessing potential digital risks in young people. This is conducted in CAMHS services across Oxford Health Foundation Trust. The results will help us design strategies to improve the ways we identify and manage these types of risks.

By "digital risks" we mean behaviours involving the use of the Internet and/or social media that poses potential risks to self or others in young people that you work with.

This audit is anonymous and individual responses would remain confidential and not shared in any form.

The audit will take around 15 minutes to complete.

If you have any questions about this audit, please contact the investigators involved:
Dr Alex Lau-Zhu, trainee clinical psychologist (alex.lauzhu@oxfordhealth.nhs.uk)
Dr Mat Lister, consultant clinical psychologist (matthew.lister@oxfordhealth.nhs.uk).

If you are interested in taking part in the next stage of this audit (e.g., interviews/focus groups; in person or remotely via teams/telephone), there will be an opportunity to leave your contact details at the end.

Please proceed to the next page to begin this survey.

SECTION I

Demographics
What is your age?
What is your gender? (Male/Female/Other/Prefer not to say)
What is your ethnic group? (Asian or Asian British [Includes any Asian background, for example, Bangladeshi, Chinese, Indian, Pakistani]/Black, African, Black British or Caribbean [Includes any Black background]/Mixed or multiple ethnic groups [Includes any Mixed background]/White [Includes any White background]/Another ethnic group [Includes any other ethnic group, for example, Arab]/Prefer not to say

Parenthood
Are you a parent? (Yes/No/Prefer not to say)
How many children do you have? (1/2/3/4/More than 4)
Please state the age(s) of your child/children:

Professional profile
What is your core professional background? (Psychiatrist/Psychologist [clinical, counselling]/ Psychotherapist/CBT therapist/CWP or EWP/Nursing/Speech and language therapist/Social worker/Occupational therapist/Other)

How many years do you have since you qualified in your core profession (0 if you have not qualified yet)?
What is your work status? (Full-time/Part-time)
What CAMHS service do you work in? (Getting Help/Getting More Help/Eating disorders/SPA/NDC/Learning and disability/Horizon/CAHBS/Forensic CAMHS/Outreach/DBT/Crisis)
How many years have you been employed in the Oxford Health Foundation Trust?

Caseload Audit

In your current role, how many direct contacts with children/young people do you do on average per week (face-to-face/digital)? (0/1-3/4-9/10-12/13-15/more than 15)
In your current role, how many contacts via family members, schools and/or team consultations do you do on average per week (face-to-face/digital)? (0/1-3/4-9/10-12/13-15/more than 15)

SECTION II

Assessment of Potential Digital Risks

In the next few pages please answer some questions about your experience on each of the 10 issues relevant to digital risks in children/young people.

Issue 1: Excessive time spent online or in social media
Issue 2: Addictive behaviours (e.g., gaming, gambling)
Issue 3: Meeting strangers online (including then meeting in the real-world)
Issue 4: Sexting
Issue 5: Abusive messages, cyberbullying or trolling
Issue 6: Exposure to sexual materials (e.g., pornography)
Issue 7: Content related to suicide or self-harm (e.g., forums, images)
Issue 8: Media traumas (e.g., viewing gruesome details of horrific events via the media)
Issue 9: Dark web (e.g., to buy illegal drugs)
Issue 10: Body image (e.g., unrealistic standards)

[Questions repeat for each issue]

How often are parents/caregivers concerned about this issue? (Often/Sometimes/Seldom/Never)
How often are the children/young people themselves concerned about this issue? (Often/Sometimes/Seldom/Never)
How often are you as a clinician concerned? (Often/Sometimes/Seldom/Never)
How often have you assessed this issue in a child/young person's (direct or indirectly via families, schools or team consultations)? (Often/Sometimes/Seldom/Never)

SECTION III

Are you aware of any resources for considering, assessing or managing digital risks in young people? (Yes/No)
If yes, from within or outside the Trust? (Within/Outside/Which one?)
In the following pages, you will see 10 different logos for a range of apps which pose varying levels of risks for children/young people. Please select the answer that best match your degree of knowledge for this app.

“Reddit”
“Kik”
“Snapchat”
“Tellonym”
"Tinder"
"Roblox"
"TikTok"
"Grindr"

[Questions are repeated for each app]

I use this personally
I have heard of it/seen it and I understand how it works
I have heard of it/seen it but I do not understand how it works
I have never heard of it or seen it
Prefer not to say
Does this app pose any risk to young people [Yes/Maybe/Don’t know]

FINAL

Next Steps

We would like to conduct interviews/focus groups in order to understand in more detail some of your views/experiences. This would greatly help us in shaping a strategy to improve how we assess and manage digital risks in children/young people across the Trust. This will not be linked to your responses in this initial survey. If you are interested, please type your email:

End

Thank you - this is the end of the survey, This following link provides a useful and up-to-date guide to digital safety for children/young people: https://www.net-aware.org.uk/

If you have any questions about this audit, please contact the investigators involved: Dr Alex Lau-Zhu, trainee clinical psychologist (alex.lauzhu@oxfordhealth.nhs.uk) or Dr Mat Lister, consultant clinical psychologist (matthew.lister@oxfordhealth.nhs.uk).

Supplemental Materials 5

Interview Topic Guide

Introductions and Warm up

- *Setting up the scene: 1) Purpose: Explore in more detail your experiences and views on digital risks in young people; 2) Confidential and anonymous; non-identifiable use of potential quotes; 3) Duration: between 30-50min*
- *Introducing interviewer: Where do you work? what is your role? What brought you to working with young people?*
- *How "digital" would you say you are? (e.g., time spent; on what)*

Core Questions

- *Definition of digital risk: behaviours involving the use of digital devices, the Internet and/or social media that pose potential harm to self, to others, or from others, in young people that you work with.*
- *What forms of digital risks are most relevant for the young people you work with?*
- *Whenever you had concerns about some form of digital risk,*
    - *Prompts:*
        - *How did you address them?*
        - *What influenced what you did ask?*
        - *What influenced what you didn't ask?*
        - *Did you always assess it? Why?*
        - *Any factors related to 1) yourself; 2) young people; 3) parents and carers, 4) your team, 5) the Trust?*
- *Do you think there is room for improvement for assessing digital risks?*
    - *Prompts:*
        - *What kind of improvements?*
        - *What needs to change?*
            - *1) Yourself as clinician? 2) Team? 3) Trust?*
        - *What would you want/need?*
        - *What would enabled you to assess digital risks?*
        - *Is there anything that is in the way? What?*
- *Do you think assessments of digital risks should be part of routine risk assessments in CAMHS?*
    - *Prompts:*
        - *Why? Or why not?*
        - *If yes: How?*
        - *What is gonna take for you to make this routine?*
        - *Are there things that would get in the way?*
- *Have there been any experiences that inform your views on digital risks?*
    - *Prompts:*
        - *Professional experiences?*
        - *Personal experiences?*

Closing

- *Any issues we haven't covered which you think it is important to share or discuss further?*
- *Do you have any questions?*

Supplemental Materials 6

Table S2

Demographics and Professional Characteristics of Clinicians Surveyed (N=53)

Age (SD; range)	*37.02 (10.11; 23-61)*
Gender	
Female	*45 (84.9%)*
Male	*8 (15.1%)*
Ethnicity	
Asian or Asian British	*3 (5.7%)*
Mixed or multiple ethnic groups	*1 (1.9%)*
White	*47 (88.7%)*
Another ethnic group	*1 (1.9%)*
Are you a parent?	
Yes	*26 (49.1%)*
No	*26 (49.1%)*
Prefer not to say	*1 (1.9%)*
Parent of children aged 12 and under	
Yes	*16 (30.2%)*
No	*36 (67.9%)*
Parent of children aged 13-18	
Yes	*10 (18.9%)*
No	*42 (79.2%)*
Core professional background	
Psychologist (Clinical, Counselling or Forensic)	*21 (39.6%)*
Nurse	*11 (20.8%)*
CWP or EWP	*5 (9.4%)*
Psychiatrist	*4 (7.5%)*
Social worker	*4 (7.5%)*
CBT therapist	*3 (5.7%)*
Occupational therapist	*1 (1.9%)*
Psychotherapist	*1 (1.9%)*
Other	*3 (5.7%)*
Years qualified since core profession (SD; range)	*8.77 (10.65; 0-39)*
Work status	
Full-time	*43 (81.1%)*
Part-time	*10 (18.9%)*
CAMHS team[1]	
Mental health support teams (Tier 2)	*3*
Getting help (Tier 2)	*5*
Getting more help (Tier 3)	*14*
Eating disorders (Tier 4)	*5*

Neurodevelopmental conditions (Tier 4)	*13*
Learning disability (Tier 4)	*2*
Young people affected by sexual harm (Horizon) (Tier 4)	*4*
Harmful behaviours (Tier 4)	*3*
Forensic (Tier 4)	*4*
Outreach (Tier 4)	*3*
DBT (Tier 4)	*2*
Crisis (Tier 4)	*1*
Others	*5*
Direct clinical contact per week	
0	*4 (7.5%)*
1-3	*17 (32.1%)*
4-9	*19 (35.8%)*
10-12	*8 (15.1%)*
13-15	*4 (7.5%)*
More than 15	*1 (1.9%)*
Indirect clinical contact per week	
0	*4 (7.5%)*
1-3	*17 (32.1%)*
4-9	*20 (37.7%)*
10-12	*6 (11.3%)*
13-15	*3 (5.7%)*
More than 15	*3 (5.7%)*
Years employed by Oxford Health (SD; range)	*6.57 (7.20; 0-31)*

Note. CAMHS = Children and Adolescent Mental Health Service; CWP = Children's Wellbeing Practitioner; EWP = Educational Wellbeing Practitioner; DBT= Dialectical Behavioural Therapy.

[1] CAMHS in the UK are organised in a tiered system including tier 2 (early help and targeted services), tier 3 (local, specialist mental health services) and tier 4 (county-wide services working with complex youth). Some clinicians work in more than one team

Supplemental Materials 7

Table S3

Exploring associations between frequency of concerns between clinicians, young people and parents/caregivers per digital risk area

Association in frequency of concerns between…	*Clinicians and young people*	*Clinicians and parents*	*Parents and young people*
time	*.665*	*<.001**	*.574*
addiction	*.277*	*<.001**	*.242*
strangers	*.072*	*.010**	*<.001**
sexting	*.001**	*<.001**	*.001**
cyberbullying	*<.001**	*<.001**	*<.001**
porn	*<.001**	*<.001**	*<.001**
suicide	*.013**	*.001**	*.002**
media	*<.001**	*<.001**	*.001**
Dark web	*.003**	*<.001**	*.064*
body	*.011**	*.019**	*<.001*

*Note. * significant after applying Benjamini-Hochberg procedure for controlling false discovery rate (q<.05)*

Table S4

Exploring associations between frequency of assessments and frequency of concerns in clinicians per digital risk area

Association in clinicians' frequency of assessment & concerns from…	*Clinicians*
time	*.004**
addiction	*<.001**
strangers	*.004**
sexting	*<.001**
cyberbullying	*.012**
porn	*<.001**
suicide	*<.001**
media	*.062*
Dark web	*.018**
body	*.005**

*Note. * significant after applying Benjamini-Hochberg procedure for controlling false discovery rate (q<.05)*

Table S5

Exploring associations between clinicians' personal characteristics and clinicians' frequency of concern per digital risk area

	Age	Parent	Children under 12	Children 13-18	Years qualified	Tiers
time	.482	.499	1.00	.668	.175	.503
addiction	.065	.160	1.00	.488	.782	.144
strangers	1.00	.382	1.00	.462	.148	.014
sexting	.544	.023	.064	.174	.002*	.003*
bullying	.736	.523	.730	1.00	.054	.187
porn	.135	.095	.551	.492	.006*	.023
suicide	.737	.541	.580	.704	1.00	.359
media	.547	.093	.365	.015	.586	.247
dark web	.442	.075	.251	.085	.175	.072
body	.252	1.00	.704	.670	.467	.708

*Note. * significant after applying Benjamini-Hochberg procedure for controlling false discovery rate (q<.05)*

Table S6

Exploring associations between clinicians' personal characteristics and clinicians' frequency of assessment per digital risk area

	Age	Parent	Children under 12	Children 13-18	Years qualified	Tiers
time	.539	.075	.055	.467	.001*	.032
addiction	.218	.264	.763	.734	.412	.247
strangers	.544	.025	.138	.296	.025*	.043
sexting	1.00	.153	.356	.156	<.001*	.017
bullying	.342	1.00	1.00	1.00	.135	1.00
porn	.209	.153	.759	.480	.001*	.240
suicide	.118	.153	1.00	.068	.024*	.771
media	.418	.140	.018	.349	.728	.458
dark web	.568	.110	.081	1.00	.051	.284
body	.539	.017	.055	.137	.008*	.360

*Note. * significant after applying Benjamini-Hochberg procedure for controlling false discovery rate (q<.05)*

Table S7

Exploring associations between clinicians' personal characteristics and clinicians' knowledge

	Age	Parent	Children under 12	Children 13-18	Years qualified	Tiers
guidelines	.351	.020*	.065	.729	.047*	.134
apps – correctly identified	.547	.781	.769	.483	.586	1.00
apps – recognised as risky	.372	.406	.132	.726	.414	.565

Note. * significant after applying Benjamini-Hochberg procedure for controlling false discovery rate (q<.05)

Supplemental Materials 8

Table S8

Themes and Subthemes and Correspondence with COM-B Components and TDF Constructs

Themes	Subthemes	COM-B component	TDF	Interventions suggested by clinicians
**1) Assessment skills**	*Information gathering*	*Capability (psychological)*	*Skills*	*Asking directly an plainly*<br>*Fit questions to age*<br>*Asking awkward questions*<br>*Semi-structured approach*<br>*Use of prompts*<br>*Ecological model*<br>*Consider protective factors*<br>*Overall risks assessment*<br>*Assess broader context*<br>*Assess general functioning*<br>*Curious and open*<br>*Use formulation to guide*<br>*Handling discrepancies in reports*<br>*Sensitive to over-questioning*
	Building relationships	*Capability (psychological)*	*Skills*	*Asking engaging questions*<br>*Using humour*<br>*Take time to get to know YP*<br>*Knowing about technology*
	Risk management	*Capability (psychological)*	*Skills*	*Understanding legalities*<br>*Advice to parents*<br>*Clear risk protocol*<br>*Supporting digital changes*<br>*Educating parents*<br>*Handling discrepancies in reports*<br>*Educating parents*<br>*Supporting parents with management*
**2) Knowledge**	*Definitions and technicalities*	*Capability (psychological)*	*Knowledge*	*Knowing all risks*<br>*Knowing mechanics*<br>*Knowing apps*
	Developmental considerations	*Capability (psychological)*	*Knowledge*	*Developmental norms*<br>*Risks mechanisms*<br>*Signs of vulnerability*<br>*Typology of risks*
**3) Mobilising resources**	*Forms and procedures*	*Opportunities (physical)*	*Environmental context and resources*	*Formal template*<br>*Include in routine reports*<br>*Collecting data pre assessment*<br>*Risk protocol*<br>*Add to Carenotes*<br>*Information accessible to clinicians*<br>*Information guide*<br>*Diagrams and flowcharts*
	Knowledge sharing	*Opportunities (physical and social)*	*Environmental context and resources; social influences*	*Brief updates*<br>*Safeguarding alerts*<br>*Share knowledge across teams*<br>*Share knowledge within teams*<br>*Training from specialist teams*<br>*Teaching during team meetings*<br>*Use business meetings*<br>*Yearly updates*

				Beware of training fatigue<br>*Email communication*<br>*Expert but empathic trainer*<br>*Upskilling via consultations*
**4) Organizational context**	*Prioritising*	*Opportunities (social)*	*Social influences*	*Agreement from management*<br>*Avoid top-down approach*<br>*Deprioritising other issues*<br>*Digital awareness week*<br>*Induction package*<br>*Awareness outside of CAMHS*<br>*Training staff on cybersecurity*<br>*Whole CAMHS on board*<br>*Embed in team discussions*<br>*Use of supervision*<br>*Digital champion*<br>*Clarity about where to go for help*
	Professional expectations	*Opportunities (social)*	*Social influences*	*National guidance*<br>*Professional guidance*<br>*Strategic framework*<br>*Knowing colleagues' practices*<br>*Ensuring everyone asks*
	Digital working	*Opportunities (physical and social)*	*Environmental context and resources; social influences*	*Continue with blended working*
**5) Empowering youth**		*Opportunities (physical and social)*	*Environmental context and resources*	*Educating young people*<br>*Digital education in schools*<br>*Clear language for young people*<br>*Not rely on self-report*<br>*Help young people to be brave to share*<br>*Risk checklist for young people*<br>*Young people's views on being assessed for digital risks*<br>*Involve young people in training*<br>*Broader communication about youth patient engagement*
**6) Professional role and identity**	*Importance of good practice*	*Motivation (reflective)*	*Social/professional role and identity*	*Promote interest in topic*<br>*Use evidence base*<br>*Regular updates/education*
	Perceived low competence	*Motivation (reflective)*	*Social/professional role and identity*	*Address generational confidence*<br>*Recognise differences in experiences*<br>*Regular updates/education*
**7) Habit change**		*Motivation (automatic)*	*Reinforcement*	*Hep build habit*<br>*Standardisation*<br>*Implement try out period*
**8) Emotional experience**		*Motivation (automatic)*	*Emotion*	*Stories of big incidents*<br>*Address taboo of sexual topics*<br>*Address fear of not knowing*<br>*Address helplessness/frustrations*

Note. COM-B = Capabilities, Opportunities, Motivations and Behaviour Model; TDF = Theoretical Domain Framework.

Supplemental Materials 9

Illustrative Quotes for Themes/Subthemes

Theme 1: Assessment skills

Information gathering

And so that's made me realise you just you can't pick it up. Normally, you've got to ask direct questions. So I've got enough clinical experience to know that asking the question 150 times until you get one Yes, that's worth asking the question for so I think culturally, I believe in that. (participant 10)

And as part of that process we go through in kind of really methodical way. So we talk about family and family experiences, and what else is this family managing and what are the other difficulties and areas of concern for that family, then how they're getting on at school, which is the biggest part of their, usually their week, when they're away from families in school, so how is school going, what's going on there for them, and then look at the behaviour that they're exhibiting which can be not just assigned to harmful sexual behaviour. (participant 12)

Building relationships

But then some of the stuff it's hard cause you wouldn't say like, I don't routinely ask people about pornography use, for example. So that only came up because the young person had been brave enough to mention it. I guess had I asked about it earlier on, I may or may not have found it, you know, so he was somebody who we've worked probably 10 or 15 sessions together, and it came out at that point. (participant 2)

You know, one particular person would be continuously refusing to come out because they were on Fortnight talking to friends, or would be trying to access it. So a lot of our conversations were about these things, because it was a barrier to the work. No, actually, you had to be really open and really interested in talking about some of those difficult conversations with them about ...why you'd want to look at this. So you'd want to look at that, "I wonder why", Perhaps "your carer doesn't think that that's such a good idea, rather than you shouldn't". It's about being really curious, isn't it? (participant 3)

Risk management

So that knowing what to do in the future would be good. Apart from just the standards things to younger, "no, but you don't have to reply". I think I did in sessions say to her, like, "don't feel that you do have to, it's okay not to" but she's not just gonna trust me, you know, when all her friends are doing that and expecting that of her ? (participant 9)

So professionals sometimes don't realise that actually, the level of verification that they need is not at the very... doesn't need to be at the very highest level...And that's pretty significant.

So you frequently get teachers or social workers or whatever saying "Oh, well, he's not going to be charged, therefore, it's not a problem". You see? it could be saying the risk here is still high on the balance of probabilities, and we still need to be doing something. (participant 6)

Theme 2: Knowledge

Definitions and technicalities

Yeah, I mean, that's very broad, isn't it? Like I suppose that would encompass things that I wouldn't, that wouldn't naturally come to my mind with digital risk, like using the internet to buy drugs and alcohol, which is definitely something that kind of young people I've worked with have done. But I interestingly wouldn't put that in like a digital risk category. (participant 1)

You can do more with "Oh, well, I know this is something you use; how are you using it? …using it in this way or that way?" Whereas if we only know for example, Snapchat, where they send pictures, but actually there's a lot more to Snapchat than just sending pictures, there is time limited, there is location things. So there's posing risks within the different apps as well. Yeah, I think so. For me, it'd be training but also some sort of interaction on how to do that or how to know about them as well. (participant 3)

Developmental considerations

I wouldn't say … that I think it's an okay thing, or that I necessarily think all of it is developmentally appropriate. But I suppose I do think some of it probably falls into the kind of experimenting, taking risk side of things that perhaps, you know, before the advent of the digital stuff we would have done in other ways, you know, teenagers or but, you know, it's just sort of translated there. And so it doesn't mean that it's okay, or that I would be accepting of it. But it would be perhaps less surprised. (participant 2)

I feel like I would have to come up with like, maybe a couple of different definitions, and depending on the age of the young person … I might need a slightly more simple. And it might be necessary to like, give some examples. So at the moment, I wouldn't be able to just give that…with some time, and … some research of my own, I could probably come up with ways to describe that. (participant 9)

Theme 3: Mobilising resources

Forms and procedures

I think unless, yeah, the fact that we don't really ask about it, or at least I don't really ask about it until a risk comes up, and then you sort of check "Is it at home?", "Is it on the devices", with follow up questions? I think the fact that it's not sort of being asked routinely, and it's not standardised. I think there's definitely a lot of room for improvement. (participant 11)

I don't know, if you're familiar with our electronic notes system Carenotes? So the risk assessment that we use on there isn't really, probably better now. But you know, it doesn't include things like digital risks, it could be better focused on young people and what the risks might be. So having it added, having something added to Carenotes about digital risks within the risk assessment that is already there. (participant 5)

Knowledge sharing

I think when new areas of safeguarding concern [are] around, I guess, ... that actually we're informed of it. I think the trickiness sometimes is closing that information loop.... there might be specific group of people who know better, but then that's not disseminated to the wider teams. So I guess it's how to make sure that communication is closed...so the awareness level continues. (participant 4)

There's a wonderful person in Oxfordshire Social Care, who has really good knowledge of everything to do with digital stuff. So understands the tech around it, understands what's going on, understands the new threats that may be there, and we kinda need that sort of person, but I don't understand that there's anyone like that in here in our Trust. (participant 12)

Theme 4: Organizational context

Prioritising

I think it's hard because I think for us as a team, it would need to be a priority of the team manager. But in order for it to be the team manager's priority, somebody ahead of her would need to have decided that it was a priority, if that makes sense, because she has lots of competing priorities and kind of information that she needs to share with us. (participant 2)

I think that there's pressure and time, on consideration of what else needs to be assessed. Obviously, in CAMHS, that initial stuff is about self-harm to self and managing risks that are associated with that. (participant 12)

Professional expectations

When you're doing other safeguarding stuff, for example, you're clear about what may or may not be needed and what, how that's thought about within a sort of strategic framework. Whereas I think it's less clear in this area, and I haven't, I mean, I'm sure it's there. but it's more piecemeal. And it's not as if it's everybody refers to one thing. (participant 6)

So if there's anything that I think we could potentially do more, is inquired more of it when we do our case consultations within our team meetings, or case discussions, 'cause we probably, within those cases, questions probably focus a bit more on the physical realm than

the digital realm, hmm. So maybe in our teams formulations, we could probably think a bit more about that as well. (participant 4)

Digital working

I think it's [remote working due to Covid] not a barrier, I think it's the opposing, is that we're very much more aware of what's going on and other people social media, so it would be updated as a team. And if something is going on Tik Tok, or if there's been a case of bullying online, so that we're more aware that that might be impacting on young people. So it's almost in the back of your mind to ask about it. (participant 3)

Well, I think there's been, it's not really around digital risk as such, but I think COVID has been a good opportunity for us to engage with young people in a preferred method for them, which is, you know, what, why would any teenager want to schlep all the way into one of our fairly grotty hospital bases, to sit in a room with a couple of other adults, when in fact, they can do it from the personal facility of their bedroom? Like they talk to their mates on a laptop….it's far more engaging and doing things in a way that they're far more used to. It's like using their language rather than our language. (participant 7)

Theme 5: Service users

Empowering young people

I know that Trust does have patient engagement. But it's not really embedded in what we do, or doesn't feel like it's in an everyday kind of way. So I know if we're doing a particular project, we can take things to patient participation. But it's not, you know, a constant conversation back and forth. And things like having that improved, might highlight things like digital risk, or what we're asking, what we're asking questions about and whether it's relevant because the young person might say, you don't ask too much about. Whereas we don't know that if we don't include children so much. And just how to structure service and so on. (participant 5)

There could be like a questionnaire that the young person fills out while they're in the waiting room, or the same with a routine outcome measures that asks about, I guess you know. cyberbullying, when asked about use of social media asked if they feel like they're vulnerable, or if they're spoken to someone that they haven't ever met before. If they know how to sort of use the internet safely, if there was a form that would ask those questions that were sent out that the young person can answer in private... Yeah, I don't know. I think I mean, that's my opinion. I think that could be a good way to go. (participant 11)

Theme 6: Professional role and identity

Importance of good practice

I didn't regularly assess fire setting because it wasn't one of the kind of things and whereas now that is like, that's just one of my, you know, depending on the referral and the thing, I have an idea of like, here's a whole bunch of questions that may or may not be relevant to you, and I do my, like, hallucinations and overvalued ideas and psychosis kind of screens, and I do my just to check you don't like threaten to kill people or break things or set fire to things and stuff. Because it's the one time in 12 that you don't think there's anything like that gonna come up and someone goes, well, actually...! you think this is why we ask everybody these questions. (participant 1)

We need to kind of meet, and we need to know, we need to be up to date with everything, we need to be up to date with technology, and what's coming out. And how, you know, how vulnerable people are using that stuff. You know, and to be aware of that the risks and the pitfalls. (participant 8)

Perceived low competence

The only thing that has up until now, maybe stopped me from asking about it is lack of awareness, which is why I think the teaching is so important to not knowing about digital risks, not knowing what to ask or what to look out for. Yes, I think just sort of lack of awareness and lack of confidence. (participant 11)

Because when you come across, because in this day and age, particularly with teenagers, I think they're ahead of their parents, when digital stuff is concerned. Because …as quick as it comes out there, that they're there because of the way they're taught it, because they start so young. They're ahead of it. So, you know… working with young people is beyond me, sometimes the digital stuff that they come up with, and I'm like, I haven't got clue what you're talking about. And I'm fairly digital (participant 8)

Theme 7: Habit change

Because it is the inertia… nobody would say, well, that's a terrible idea, I don't want to do that. But it's moving from….It's a bit like any kind of habit change in normal life. Isn't it moving from... "That's a good idea, like, yes, drink more water and eating more fruits and vegetables. Those are good ideas. I should do that" to "I'm going to actually put something in place." That means that I do that on a regular basis, rather than like, yeah, January or like when it's mentioned in the referral form. (participant 1)

I think that is a barrier, potentially, that it's just another learning thing. And it gets to the back of your mind if you're not continually practising it, or talking about. (participant 3)

Theme 8: Emotional experience

…a young person basically wrote this, like sort of speech to that school, and said that, you know, it was basically saying … that she felt that young people…we're so addicted to social media, and even though…everything that they teach in school is really important. … they

don't teach that. And I remember, she wrote it to her teacher, and her teacher sent it to me, because he thought that I might be able to help and I didn't know what to do. So that was an example where if I'd had some resources or something, I would have been able to help, but I just kind of didn't really know what to do. And I never been trained in like, how to how to help with that. ... I guess that made me realise that I just didn't really know what to do about that. Yeah, made me feel helpless in that way. (participant 9)

People get very worried because that sort of taboo areas to some extent, they get very worried about making judgments on the basis of, of their own sense of something rather than on some sort of external, legal or other criteria. (participant 6)

Supplemental Materials 10

Table S9

Summary of Targets for Behavioural Change and Potential Approaches

Determinants	*Intervention functions*					*Levels of intervention*			*Policy categories*			
	Education	*Training*	*Modelling*	*Environmental restructuring*	*Enablement*	*Staff*	*Teams*	*Organization*	*Guidance*	*Regulation*	*Communication*	*Environmental planning*
Assessment skills (C)		*X*				*X*			*X*	*X*		
Knowledge (C)	*X*					*X*			*X*	*X*	*X*	
Mobilising resources (O)				*X*			*X*	*X*	*X*	*X*		*X*
Organisational context (O)			*X*		*X*			*X*		*X*	*X*	
Empowering youth (O)					*X*		*X*	*X*	*X*	*X*	*X*	
Habit change (M)		*X*			*X*	*X*	*X*			*X*	*X*	*X*
Emotional experience (M)		*X*	*X*		*X*	*X*	*X*				*X*	
Professional role & identity (M)	*X*		*X*			*X*	*X*		*X*	*X*	*X*	

Notes. C = capabilities; O = opportunities; M = motivations. See Supplemental Materials 1 for brief description of intervention functions and policy categories
